# Smoking cessation assistance among pneumologists and thoracic surgeons in Switzerland: a national survey

**DOI:** 10.3389/frhs.2024.1420277

**Published:** 2024-09-18

**Authors:** Fabrizio Minervini, Peter Kestenholz, Frank Rassouli, Susanne Pohle, Nora Mayer

**Affiliations:** ^1^ Division of Thoracic Surgery, Cantonal Hospital of Lucerne, Lucerne, Switzerland; ^2^ Lung Center, Cantonal Hospital St.Gallen, St.Gallen, Switzerland

**Keywords:** smoking cessation, lung cancer, national survey, prevention, NSCLC

## Abstract

**Objective:**

Smoking, with a prevalence of about 25%–30% in Switzerland, is proven to cause major systemic, avoidable diseases including lung cancer, increasing societies morbidity and mortality. Diverse strong quitting smoking recommendations have been made available providing advice facilitating smoking cessation globally. In other European countries like Germany, clinical practice guidelines for smoking cessation services have been implemented. However, in Switzerland, there is still no national consensus on a comprehensive smoking cessation program for lung cancer patients nor on the adequate provider. Our primary aim was to assess the current status of smoking cessation practice among specialists, mainly involved in lung cancer care, in Switzerland in order to uncover potential shortcomings.

**Material and methods:**

A self-designed 14-items questionnaire, which was reviewed and approved by our working group consisting of pneumologists and thoracic surgeons, on demographics of the participants, the status of smoking cessation in Switzerland and specialists' opinion on smoking cessation was sent to thoracic surgeons and pneumologists between January 2024 and March 2024 via the commercially available platform www.surveymonkey.com. Data was collected and analysed with descriptive statistics.

**Results:**

Survey response rate was 22.25%. Smoking cessation was felt to positively affect long term survival and perioperative outcome in lung cancer surgery. While 33 (37.08%) physicians were offering smoking cessation themselves usually and always (35.96%), only 12 (13.48%) were always referring their patients for smoking cessation. Patient willingness was clearly identified as main factor for failure of cessation programs by 63 respondents (70.79%). Pneumologists were deemed to be the most adequate specialist to offer smoking cessation (49.44%) in a combination of specialist counselling combined with pharmaceutic support (80.90%).

**Conclusion:**

The development of Swiss national guidelines for smoking cessation and the implementation of cessation counselling in standardized lung cancer care pathways is warranted in Switzerland to improve long-term survival and perioperative outcome of lung cancer patients.

## Introduction

Smoking tobacco is the greatest avoidable risk factor for multiple disorders including pulmonary and cardiovascular disease and increases postoperative morbidity and mortality (1, 2). From adulthood, smoking individuals are likely to lose around three months of their lifespan for every additional year of smoking (3). Moreover, cigarette smoking is the most important modifiable risk factor for lung cancer by far, causing about 2/3 of all lung cancer cases worldwide (4). Lung cancer remains the third most common type of cancer worldwide and accounts for the highest number of cancer-related deaths in Europe (5). In Switzerland, the current smoking prevalence in the general population is 27%. Males are reported to have a higher prevalence of 31% than females 23.3% (6). Unfortunately, most lung cancer candidates qualifying for pulmonary resection are previous or current smokers (7).

Especially in surgical lung cancer candidates, quitting smoking at any time, even postoperatively, was reported to be valuable in reduction of perioperative morbidity and mortality and improve progression free survival (8–14). The prognosis of patients with lung cancer has even shown to depend on the smoking status (10, 15). In addition, even though there are no reassuring prospective data published yet, a longer period of smoking cessation improved the surgical outcome of lung cancer surgery patients (9). Going a step further into lung cancer therapy, quitting smoking also reduced the rate of infection and radiation pneumonitis during radiotherapy and prolonged the median survival after chemoradiotherapy for small-cell lung cancer (16).

Although many people who smoke are aware of their self-destructive behaviour and the majority want to quit, unassisted smoking cessation attempts fail in 95% of cases within one year due to various reasons including physical and psychological withdrawal symptoms (17). Self-reported smoking abstinence rates associated with a comprehensive quitting smoking program, however, have yielded in about 45% in a prospective, non-randomized study including 3,245 smokers with current cancer or cancer survivor treated at a comprehensive cancer centre (18).

A combined cessation approach including pharmacotherapy and behavioural interventions achieves most effective abstinence rates, which is reflected in the German S3 clinical practice guidelines for smoking cessation (19–22). To support patient decision, a recent investigation of Reinhardt et al. has shown, that pharmacotherapy to support quitting had no negative side effects on patients even when having chemotherapy in parallel (23). However, quit smoking programs are not generally provided according to evidence-based methods and physician uptake on providing smoking cessation counselling is rather low (24). Moreover, it is well known that success-rates of smoking cessation are much better if individuals participate in evidence-based smoking cessation programs with reimbursement of the costs (25). This plays a particularly important role, as it is known that lower socioeconomic status is associated with higher rates of smoking (26).

In Switzerland, to date, there is no national consensus on generally applicable guidelines for smoking cessation available, leading to highly heterogenous approaches to the afore mentioned. Smoking cessation counselling is furthermore often outsourced to non-medical providers.

In November 2023, a Swiss working group was established with the aim to investigate the smoking cessation practices among thoracic surgeons and pneumologists in Switzerland who work hand in hand in the treatment of lung cancer patients. As quitting is of high importance in view of the devastating health effects of tobacco consumption, we identified a need to conduct a survey aiming to assess the current situation in Switzerland in view of potentially changing the Swiss landscape of smoking cessation, as a matter of fact, mainly in lung cancer patients. The results of this survey are described in this article.

## Material and methods

Using the national registry of the Swiss Society for Thoracic Surgery and Swiss Society for Pulmonology, we identified 400 participants among doctors most commonly involved in lung cancer care (thoracic surgeons and pneumologists). They were invited to complete a 14-item survey to assess the current practice of smoking cessation in Switzerland. The survey was distributed electronically via Survey Monkey (©www.surveymonkey.com) in January 2024 with anonymous response collection of all active members of each organization. The introductory sentence of the survey on the Survey Monkey platform addressing the participants was “Smoking cessation in Switzerland among specialists involved in lung cancer care—a national survey designed by pneumonologists and thoracic surgeons”. All potential respondents received at least two reminder emails at 2-week intervals, with survey closure in March 2024. The questions were designed to retrieve objective data on the participants demographics and professional status, their experience with smoking cessation and their opinion on smoking cessation programs in lung cancer patients. The items were structured in different types of questions with closed questions, typical five-point Likert items to strongly agree through strongly disagree or always—never with a given statement and one feedback questions. All data were collected into a database, with descriptive data summarized as frequencies with absolute numbers and percentages. Tests of statistical significance were not conducted for every single item, given that the number of possible comparisons was too high to report relevant conclusions. A subgroup analysis was not possible due to the small number of participants. Data were collected prospectively and analysed using the SPSS statistical software programme version 20.0 for Windows (©SPSS, Chicago, IL, USA). Ethics committee approval was not required due to the absence of patients' data. Implied consent was presumed based on participant's voluntary and anonymous responses.

## Results

Eighty-nine participants responded (22.25% response rate) in total of which 36 were thoracic surgeons (40.45%) and 53 pneumologists (59.55%). Email delivery rate reported by the survey platform was 100%. Item completion rate was 100%.

### Demographics

Responders were quite evenly distributed between different ages group. Thirteen (14.61%) of them were between 25 and 34 years old, 42 (47.19%) between 35 and 44, 25 (28.09%) between 45 and 54 and 9 (10.11%) above 55 years old. 62.92% were male, 37.08% female. 19 participants (21.35%) were still in training, whereas 28 (31.46%) were in their first and 27 (30.34%) in their second decade as consultant specialist. Only 15 (16.85%) had been practising for more than 20 years. Forty-three (48.31%) were currently based in academic institutions, whereas 25 (28.09%) were working in non-academic hospitals while 15 (20.27%) were practicing in a private practice and 6 (6.74%) in a private hospital.

### Smoking cessation practice

**Figure 1:**
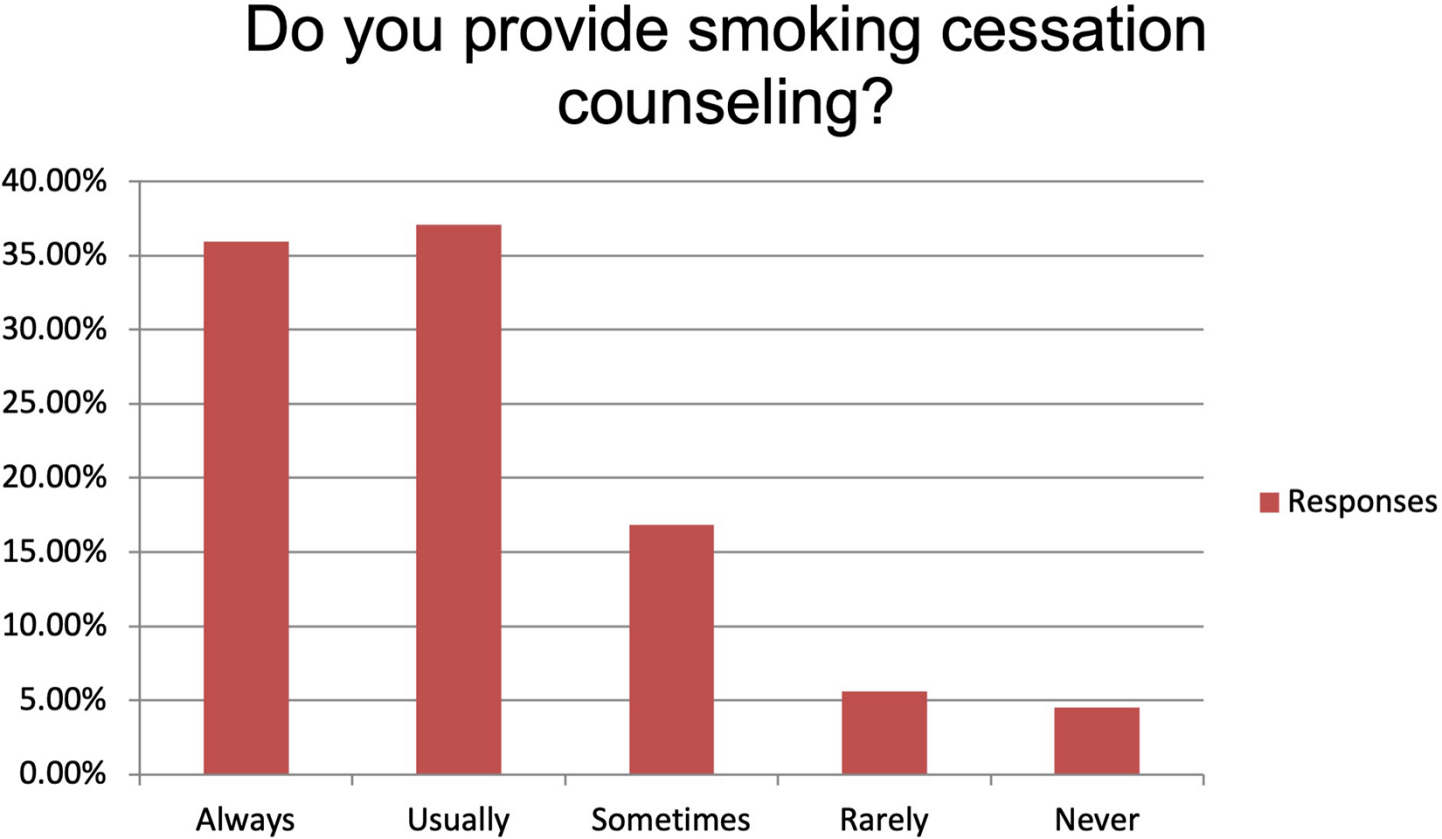
Practices about smoking cessation counseling.

**Figure 2:**
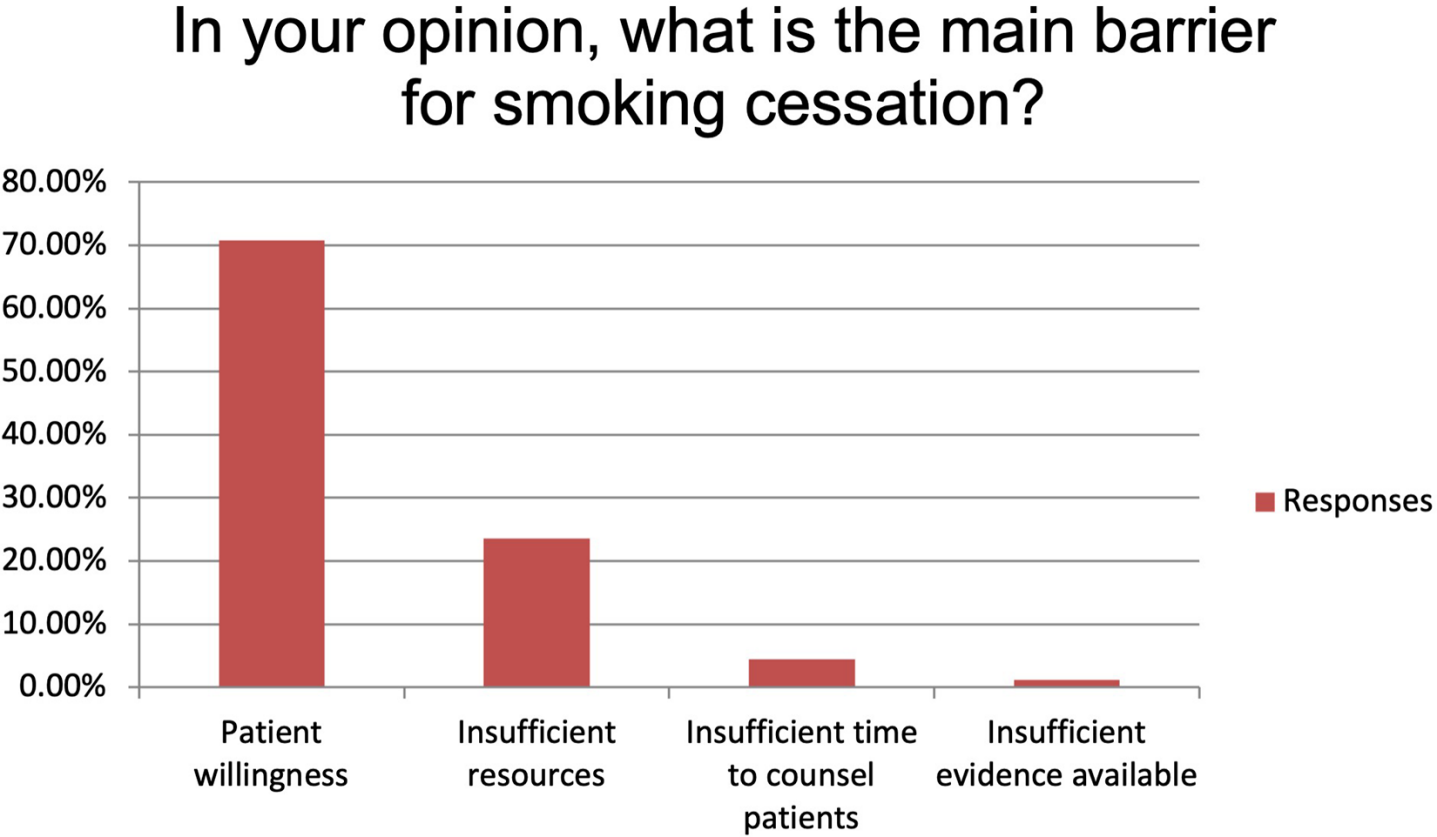
Identification of main barrier for smoking cessation.

**Figure 3:**
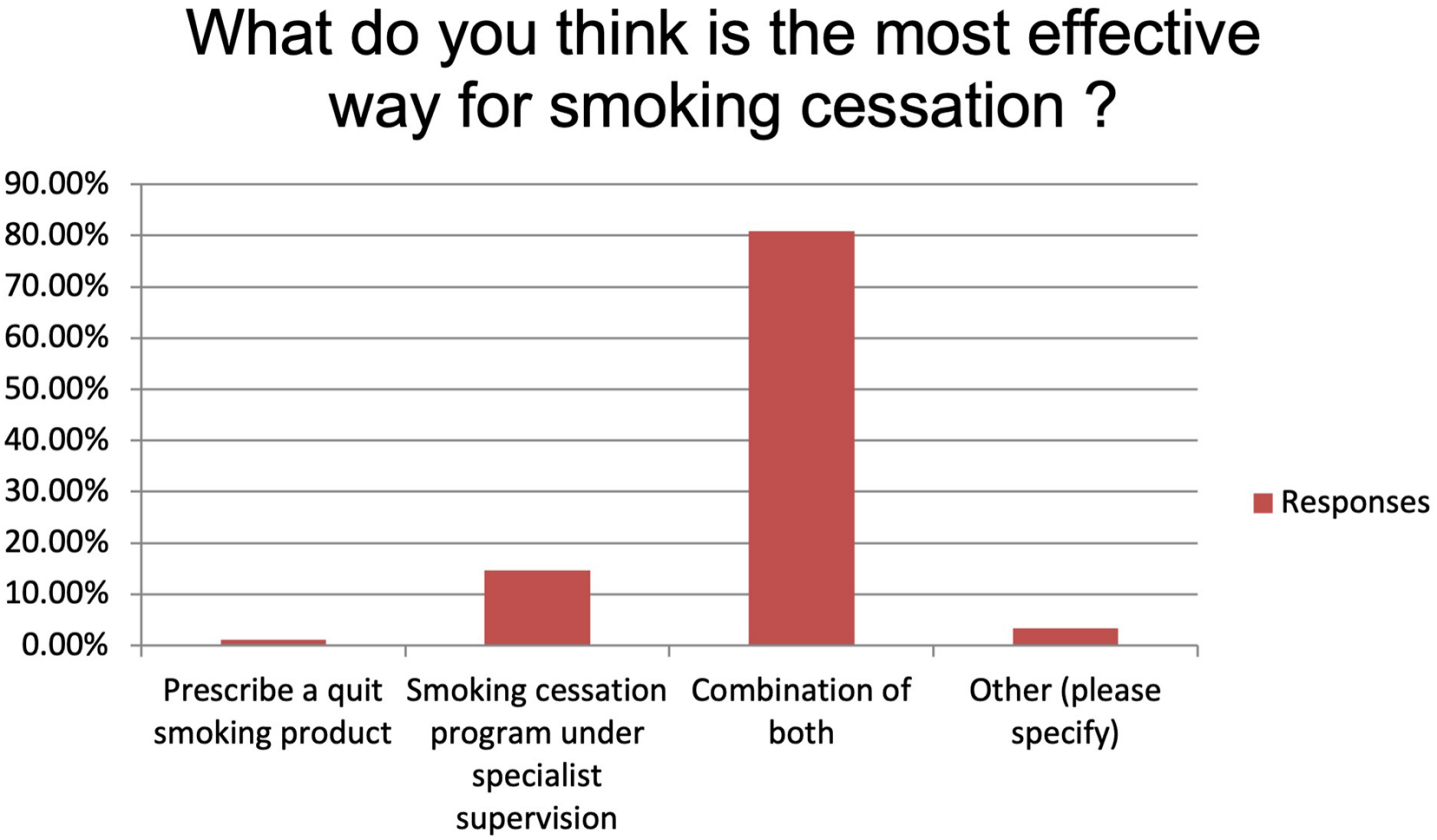
What do you think is the most effective way for smoking cessation?

**Figure 4:**
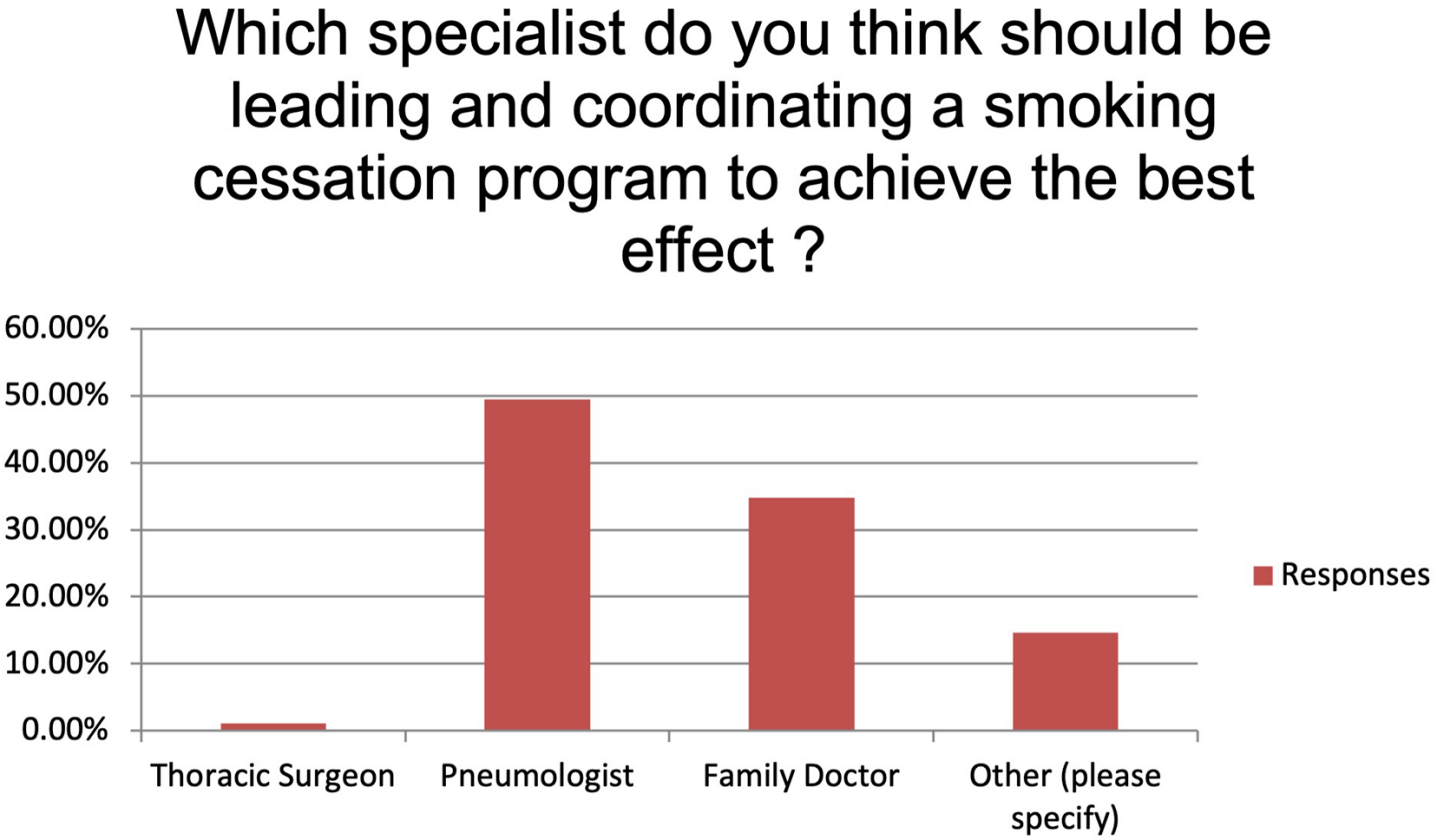
Who should coordinate a smoking cessation program?

**Figure 5:**
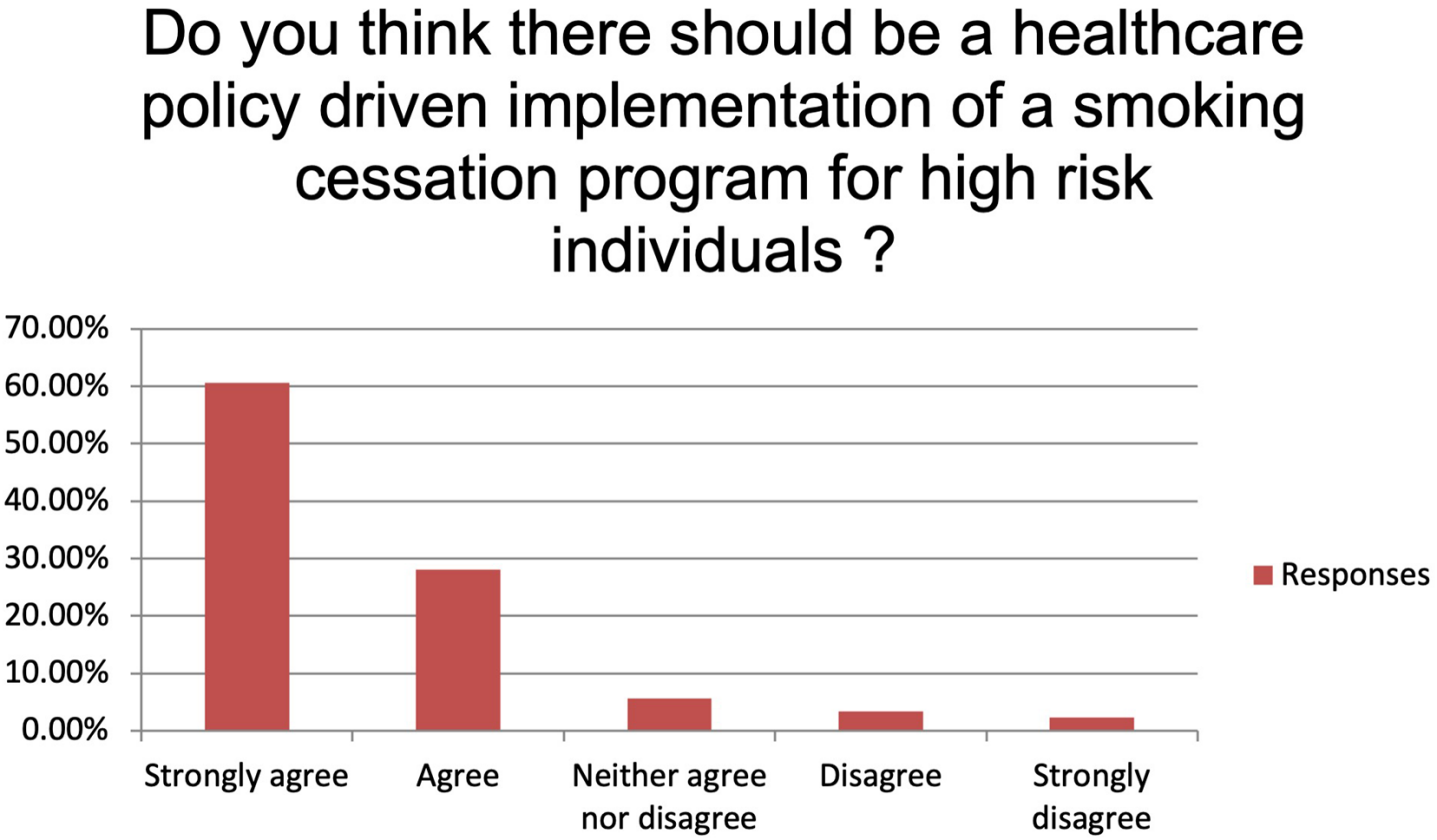
Do you think there should be a healthcare policy driven implementation of a smoking cessation program for high-risk individuals?

The majority of the respondents state to always or usually provide smoking cessation counselling (respectively 35.96% and 37.08%). Fifteen participants (16.85%) provide smoking cessation counselling sometimes while five (5.62%) and four (4.49%) only rarely or never (Figure 1). Main reported reasons for not providing it were language barrier (15%), lack of interest (31%), poor knowledge (28%) and shortage of resources (26%). Despite this, 89.19% of the participants believe that a smoking cessation program could improve the peri-operative outcomes significantly (n: 40, 44.94%) or moderately (n: 36, 40.45%) and 95.51% (n: 81) feel it would improve the long-term survival. More than 70% (n: 63) of the participants identify patient's willingness as the main barrier to smoking cessation while insufficient resources and insufficient time to counsel patients account for 23.60% and 4.49% of the answers, respectively (Figure 2). Eighty-one percent of the respondents think that combining a prescription of a quit smoking product together with an ongoing specialist supervision is the most effective way to provide smoking cessation counselling. Only a minority thinks that a supervision (meaning counselling and follow-up) by a specialist alone would be enough (n: 13, 14.61%) or only the prescription of a quit smoking product (n: 1, 1.12%) (Figure 3). When asked who the leader and coordinator of a smoking cessation program should be, 44 (49.44%) answered a pneumologist while 31 (34.83%) think that the family doctor should be responsible and 13 (14.61%) believe that a dedicated team including psychologists and trained nurses should be created (Figure 4). Lastly, a smoking cessation program should be implemented by a driven healthcare policy for most of the respondents (60.67% strongly agree, 28.09% agree) while 3.37% and 2.25% disagree and strongly disagree with that statement (5.62% are neutral about it) (Figure 5).

## Discussion

This survey highlights the importance of smoking cessation in lung cancer care, in order to improve mid- and long-term outcomes. In the presence of the obvious awareness of the positive impact on overall survival as well as disease-free survival in lung cancer, it is surprising how little has been done so far to improve the availability of smoking cessation in this special lung cancer patient cohort in Switzerland.

Most of this survey's participants believe that patient factors (lack of willingness) are the main barrier interfering with their ability to counsel patients to quit smoking as previously reported as well in a US-American survey amongst thoracic surgeons and another study amongst members of the International Association for the Study of Lung Cancer (IASLC) (27, 28). However, Babb et al. have previously shown that patient willingness to stop smoking was around 68% in adults ≥18 years in North America, and exceptionally high in lung cancer patients with close to 90% expressing the wish to quit in the admittedly small study of Gemine et al. in 2017 (29, 30). We assume, that the lack of funding for smoking-cessation interventions in Switzerland might partially explain patients’ unwillingness to engage in quit smoking counseling. A survey amongst lung cancer patients bringing light to patients’ motives, revealing the reasons for the assumed “unwillingness” to quit, will be another interesting project arising from our current assessment on the medical provider side. Understanding patient barriers to engage in quitting needs to be more thoroughly investigated to subsequently improve our strategies achieving better quit rates. We identified studies reporting very heterogenous barriers amongst individuals who smoke like e.g., “social cohesion”, “enjoyment”, “cravings”, “stress relieve” and “not being scared of consequences” (31–34).

Murray et al. just recently demanded the clarification of the funding situation to better support patients in their decision making (35). Funded programs like the tobacco treatment program at The University of Texas MD Anderson Cancer Center yielded in a 45% abstinence rate among participants (36). Even though the abstinence rate was self-reported and not biochemically verified, this example could be a model to be adapted in other countries like Switzerland.

The participants in the above-mentioned funded program at the MD Anderson Cancer Center were regularly counselled for a period of 8–12 weeks and received individualized, tailored treatment including pharmaceutical and behavioral support. The program was funded (cost per quitting individual was about $1,900–$2,500/case) through the State of Texas Tobacco Settlement Funds, making it free of cost for the smoking individuals. However, current trends in several countries were showing authorities cutting down on smoking cessation budgets (37, 38).

The question has been debated many times, whether hospitalized patients acutely affected by their lung cancer diagnosis are more susceptible to quit smoking advice, “exploiting” the “teachable moment” in patients’ favor. Other authors worry, at the time of diagnosis, patients may not recognize the beneficial effect of quitting when already diagnosed with lung cancer (14). Moreover, a crucial point is if it is ethical to postpone, or even cancel surgery, considering the influence of smoking on peri- and postoperative complications and consequently the burden on healthcare resources. Although debatable, 47% of cardiothoracic surgeons responded yes to the question “Are there any procedures that you will not perform on patients who are daily smokers” in a survey conducted in the USA. The procedures that the surgeon would avoid performing in patients who are currently smoking were: pneumonectomy (77%), open lobectomy (56%) and esophagectomy (56%) (39). Even Hippocrates with his sentence “before you heal someone, ask him if he's willing to give up the things that make him sick” seems to confirm the above-mentioned surgeons' point of view.

One more controversial topic is represented by the period of smoking abstinence that should be suggested to all the smoking patients undergoing surgery. Contrasting results have been published and so far, no consensus exists. Møller and colleagues reported that 6–8 weeks before a surgical procedure (hip and knee replacements in his trial) was sufficient to decrease wound complications, cardiovascular complications or the need for redo surgery (40). In the field of thoracic surgery, contradictory results have been reported. Barrera and colleagues did not find a different rate of pulmonary complications between patients who quit smoking >2 months before surgery and patients who quit smoking <2 months before and patients who were still smoking in the immediate preoperative period (41). Nakagawa reported an important finding, namely that the risk of postoperative pulmonary complications was decreased after a smoking abstinence of at least 4 weeks pre-operatively (42). A retrospective analysis published by Shigeeda and coll. extended to a minimum of 6 weeks of smoking cessation in order to have a reduced rate of pulmonary complications (43). In our survey we did not deal with this specific topic because it will be part of a separate future project by our study group designed for thoracic surgeons.

There is unfortunately no prospective study available comparing the difference in susceptibility of patients approached with smoking cessation intervention while undergoing lung cancer treatment as compared to current smokers without having a lung cancer diagnosis. Recruiting for such a study could potentially be done during lung cancer screening.

Apart from patient willingness negatively influencing the approach to smoking cessation counseling, some survey participants moreover believed that insufficient medical resources or time may play an important role in supporting patients. These results are again similar to those reported in the paper of Marrufo et al. and the IASLC members (27, 28). We did not further define the exact setting of “counselling” in our questionnaire, because it is widely known in Switzerland, that neither the thoracic surgery nor the pneumology departments have resources at their disposal for separate smoking cessation appointments for their lung cancer patients. Cessation counselling is therefore commonly integrated into the clinic appointments.

The mentioned language barrier keeping physicians from providing smoke-stop support is not an issue restricted to cessation itself but the whole doctor-patient relationship and should be tackled in a bilateral approach. The patient ought to be accompanied by a person to help translation while the medical provider should have easy access to a financially supported translate line service 24/7.

Outsourcing quit smoking programs to a secondary institution like currently done in some Swiss cantons e.g., to the Lungenliga (www.lungenliga.ch), the Swiss Association for tobacco control (www.at-schweiz.ch) or to Lunge-Zürich (www.lunge-zuerich.ch) may be a solution to overcome time constraints felt by physicians.

Another option to overcome the shortage of medical resources for cessation counselling might be an alternative continuous motivational support with mobile phone-based messaging or web-app based interventions to support quitters (44, 45).

Probably, the peculiarity of the Swiss health care system, being highly decentralized and with mandatory health care insurances, may play another role. The high degree of autonomy of the cantonal authorities combined with the presence of many different healthcare insurances, lacking a uniformity in health cost coverage, creates a very heterogenous healthcare landscape. A more transparent and easily accessible, uniform stop smoking program throughout all Swiss cantons might be a start to improve the smoking abstinence rate in Switzerland. 90% of the survey respondents moreover supported the implementation of a national healthcare policy driven cessation program.

Besides lack of willingness of the patients’, survey participants admitted that on the doctor's side, poor knowledge and expertise on cessation practice was a reason not to provide quit smoking counselling. Even for us as a Swiss specialist working group comprising pneumologists and thoracic surgeons, complex background research on cessation-aid pharmaceuticals approved on the Swiss market had to be done. In addition, we had to contact the umbrella organisations of the Swiss healthcare insurances (Santésuisse and Curafutura) to receive profound and complete information on the availability of financing models in the Swiss healthcare landscape.

In Switzerland, the following pharmaceutical products are registered for smoking cessation: nicotine replacement (patches, chewing gum, tablets, oral spray, inhaler), Varenicline and Bupropione. The costs for nicotine replacement products are generally not covered by Swiss health insurances. For Varenicline and Bupropione, the possibility of covering the costs once in an 18-month-timeframe exists, when certain criteria are fulfilled: the diagnosis of nicotine dependency according to the “Diagnostic and Statistical Manual of Mental Disorders” (DSM-IV) or the “International Classification of Diseases” (ICD-10) and a score of ≥6 in the test of Fagerström or the diagnosis of a smoking-induced disease. The current first-line recommendation is the combination of Varenicline or a combined long- and short-acting nicotine-replacement with ongoing counselling.

Perhaps, greater success with tobacco cessation programs would be linked to a more holistic approach to the complex patient situation considering that a recent study demonstrated that cancer stage, comorbidities, psychiatric disease, type of insurance, level of education, and emotional support were predictors for successful tobacco cessation (46, 47). This more complete patient approach factor matches well with most of our survey participants demanding, that pneumologists and family doctors should preferably take the lead in smoking cessation programs rather than the thoracic surgeon, who mainly just touches upon the patient's journey for an oncological resection. Nonetheless, the surgeon should not shirk responsibility but consistently point out existing options and always refer patients to the above-mentioned specialists for cessation counselling. The reported awareness of the importance of tobacco cessation internationally among physicians who care for lung cancer patients is high, however, data of the IASLC study suggested, that physicians do not feel adequately prepared to deliver effective tobacco cessation support (28). This fact again matches with our survey results of participants naming lack of knowledge on adequate smoking cessation support as a factor for not providing a cessation program. Previous studies have shown that e-learning or web-based continuing medical education (CME) programs were an effective way to educate healthcare providers in smoking cessation treatment (48). Moreover, Pbert et al. for example, published the recommendation for a national training and certification program (in a US-American setting), however they mention the challenge of time constraints in provider education (49, 50). Healthcare specialist education could even start early in a doctor's journey in their residency as described by Ockene et al. to raise awareness and create a basis of knowledge (51).

Given the positive effects on short- and long-term outcome of tobacco abstinent lung cancer patients, we must claim smoking cessation to be an inconceivable part of lung cancer treatment (52). Smoking cessation counselling could potentially be combined with a lung cancer screening with low-dose CT chest consultation. As known from the NELSON-trial, the evidence is growing that lung cancer screening reduces lung-cancer related mortality, as does quitting tobacco consumption (53). In the United States and the United Kingdom, as well as in a few European countries, first lung cancer screening programs have already been established. Jungblut et al. just recently published their investigations on the feasibility of a Swiss screening program called “The Swiss Approach” in 2022. The authors as well recommend offering additional smoking cessation for individuals selected for screening (54).

Other than felt by the survey participants, the majority of lung cancer patients are willing to quit tobacco consumption (55). However, abstinence success rates without a specialist guided cessation program on the contrary were rather low. As the awareness of the importance of smoking cessation in physicians involved in lung cancer care is high, we ought to improve the status of education on smoking cessation amongst physicians. In this way, one could strengthen their ability in providing better support for our lung cancer patients, as apparently, a doctor's advice still is an important patient perceived reason for smoking cessation (56).

## Strengths and limitations

One strength of this survey is the response rate of around 22% obtained, given that it is not unusual for healthcare related surveys to achieve response rates below 20% (57). Moreover, we also included a free-text answer field (Question 7, see questionnaire in Supplementary Material) to obtain accurate details and allow respondents to specify their responses with pertinent details.

Possibly, this survey could have been negatively influenced by the social desirability bias, namely the tendency of survey participants to respond to questions in a way that will be judged as favorable (58). Another limitation is, that the questionnaire design was not led by a Delphi process, but a specialist committee was creating, evaluating and approving the questions. We did not conduct a pilot study to confirm the accuracy of the questionnaire.

Furthermore, due to the small sample size, we were not able to perform subgroup analyses evaluating differences in answers based on geography, discipline, or years of experience in practice.

## Conclusion

The importance of a unified and accessible smoking cessation program in Switzerland is well recognized by physicians. However, no generally available Swiss guidelines on recommended practice are available. Derived from this survey, we strongly recommend the development and approval of Swiss national guidelines for smoking cessation, the improvement of the availability of physician education possibilities on available cessation interventions and the implementation of cessation counselling in standardized lung cancer care pathways. Uniform funding opportunities, e.g., implemented in each Swiss health care insurances optional (prevention) packages to make cessation affordable for individuals who want to quit for agreeable cost, should be approved through health care officials to optimize patient engagement in smoking cessation and improve overall outcomes in lung cancer care.

## Data Availability

The raw data supporting the conclusions of this article will be made available by the authors, without undue reservation.
