# Supplementary material for: Smoking cessation assistance among pneumologists and thoracic surgeons in Switzerland: a national survey
Source: Front Health Serv. 2024 Sep 18;4:1420277. doi: 10.3389/frhs.2024.1420277 (PMC11445225; doi:10.3389/frhs.2024.1420277)
Supplement: Supplementary file 1 [file Table1.docx]

**Introduction**

Dear colleagues,

We're running a survey and would love your input. Please let us know what you think below.
The survey contains 5 general questions concerning your specialty followed by 9 questions on smoking cessation. The results of the survey will help us to understand the *status quo* in Switzerland in order to establish a national standard smoking cessation program.
Thank you for participating!

**Survey**

1) What is your current gender identity?

Woman

Man
Non-binary

Transgender
Prefer not to disclose

2) What's your age?

25-34

35-44

45-54

>55

3) What is your specialty?

Thoracic Surgery

Pneumology

4) How many years of experience do you have in your field?

<5 years

5-10 years

10-20 years

>20 years

5) Where are you working right now?

University hospital

Non university hospital

Private hospital

6)Do you provide smoking cessation counseling?

Always

Usually

Sometimes

Rarely

Never

7) If you are not providing smoking cessation always, which are the reasons? (Open question, please comment)

8) How do you envision patients perioperative outcomes for major lung surgery being improved with a smoking cessation program ?

Significant improvement

Moderate improvement

Neutral

Slight improvement

No improvement

9) How do you think current smokers long term survival will be improved with a smoking cessation program?

Significant improvement

Moderate improvement

Neutral

Slight improvement

No improvement

10) How often you refer your patients to smoking cessation programs?

Always

Usually

Sometimes

Rarely

Never

11) What is in your opinion the main barrier to smoking cessation?

Patient willingness

Insufficient resources

Insufficient time to counsel patients

Insufficient evidence available

12) What do you think is the most effective way for smoking cessation:

Prescribe a quit smoking product

Smoking cessation Program under specialist supervision

Combination of both

Other (please specify)

13)Which specialist do you think should be leading and coordinating a smoking cessation program to achieve the best effect

Thoracic surgeon

Pneumologist

Family Doctor

Other (please specify):________

14) Do you think there should be a healthcare policy driven implementation of a smoking cessation program for high risk individuals ?

Strongly agree

Agree

Neither agree nor disagree

Disagree

Strongly disagree
